# RETRACTION: Clinical Observation of Ultraviolet Therapy Combined with Autologous Platelet‐Rich Plasma in the Treatment of Chronic Refractory Wounds

**DOI:** 10.1111/iwj.70165

**Published:** 2024-12-15

## Abstract

**RETRACTION:** LiG. , LiuS. , TanG. , WangS. , and XieJ. , “Clinical Observation of Ultraviolet Therapy Combined with Autologous Platelet‐Rich Plasma in the Treatment of Chronic Refractory Wounds,” International Wound Journal 21, no. 4 (2024): e14746, 10.1111/iwj.14746.38654547 PMC11040179

The above article, published online on 23 April 2024, in Wiley Online Library (http://onlinelibrary.wiley.com/), has been retracted by agreement between the journal Editor in Chief, Professor Keith Harding; and John Wiley & Sons Ltd. Following an investigation by the publisher, all parties have concluded that this article was accepted solely on the basis of a compromised peer review process. In addition, the investigation found there was significant unattributed textual overlap between this article and a previously‐published article (Liao et al. 2020 [https://doi.org/10.1016/j.jss.2019.09.019]). The editors have therefore decided to retract the article. The authors did not respond to our notice regarding the retraction.

**RETRACTION:** G. Li , S. Liu , G. Tan , S. Wang , and J. Xie , “Clinical Observation of Ultraviolet Therapy Combined with Autologous Platelet‐Rich Plasma in the Treatment of Chronic Refractory Wounds,” International Wound Journal 21, no. 4 (2024): e14746, 10.1111/iwj.14746.38654547 PMC11040179

The above article, published online on 23 April 2024, in Wiley Online Library (http://onlinelibrary.wiley.com/), has been retracted by agreement between the journal Editor in Chief, Professor Keith Harding; and John Wiley & Sons Ltd. Following an investigation by the publisher, all parties have concluded that this article was accepted solely on the basis of a compromised peer review process. In addition, the investigation found there was significant unattributed textual overlap between this article and a previously‐published article (Liao et al. 2020 [https://doi.org/10.1016/j.jss.2019.09.019]). The editors have therefore decided to retract the article. The authors did not respond to our notice regarding the retraction.

